# Supplementary material for: Learning curve and surgical outcome of robotic assisted colorectal surgery with ERAS program
Source: Sci Rep. 2022 Nov 29;12:20566. doi: 10.1038/s41598-022-24665-w (PMC9709162; doi:10.1038/s41598-022-24665-w)
Supplement: Supplementary file 2 — Supplementary Information 2. [file 41598_2022_24665_MOESM2_ESM.zip › RAL Raw data-Ñ[▒K/071/HS_ñw╝╨░O▒Kñσ.pdf]

正式摘要(電子簽章：000000214282952)
W55 / W55 ☐ 英文標題(注意:無英文姓名-請補病人基本檔!) ☐【讀取】憑證

臺中榮民總醫院
出院病歷摘要

轉入號： 轉出號：

[redacted]

(8)流水編號	
(9)入院日期	110/04/07 (2021) 直腸外科 W55 - 242
(10)轉科日期	
(11)出院日期	110/04/12 (2021) 住院天數計 5 日

診斷
- 入院 :1. Malignant tumor of sigmoid colon
  2. Subtotal gastrectomy with B-II reconstruction, 41 YEARS AGO IN DR KOO s/p biopsy at anatomosis site
- 出院 :1. Ca of R-S colon with partial obstruction, sT4N1M0, s/p robotic anterior resection on 2021/04/08
  2. Subtotal gastrectomy with B-II reconstruction, 41 YEARS AGO IN DR KOO s/p biopsy at anatomosis site

主訴
- for anterial resection

病史
- This 90 year old male had past hisotry with
  1.Subtotal gastrectomy with B-II reconstruction, 41 YEARS AGO IN DR KOO s/p biopsy at anatomosis site
  2.Hypertension under medication control.
  3.BPH under medication control.
  He suffered from diarrhea for 4 months, and had no response to anti-diarrhea drugs. Body weight loss 3 kg in rencent 3 months also was noted. He went to our GI OPD, and UGI+LGI scope were arranged. Sigmoid colon, 25 cm from anal verge, "C", Adenocarcinoma, moderately differentiated was found. He had no fever, no anorexia, no nausea, no vomitting, no abdominal pain. This time, he was admitted for surgery intervention.

體檢發現
-
  - General appearance :
    - easy looking , conscious clear , without cardiopulmonary distress
    - Vital signs自動帶入date 體溫(BT) 脈搏(P) 呼吸(R) 血壓(BP) 身高(BH) 體重(BW) BMI
      20210407 36.1℃ 50-次/分 18-次/分 128/75mmHg 167.9cm 65Kg 23.1
    - Vital signs: B.P. 128/75 mmHg , P.R. 50 /min , R.R. 18 /min , B.T. 36.1 ℃
  - HEENT :
    - Eyes: no anemic conjunctiva, no icteric sclera, normal EOM
    - ENT: no otorrhea, no rhinorrhea, no erythematous mucosa, no injected tonsil, no deformity
  - Neck: supple, freely movable, trachea midline,no deformity, no engorged jugular vein , no palpable thyroid or lymph node enlargement
  - Chest:
    - Inspection: Grossly normal, symmetric and fully expansion, no accessory muscle used, no operation scar, no spider angiomata, no gynecomastia
    - Palpation: no palpable mass or nodule, no subcutaneous emphysema, no tenderness
    - Ausculation: clear breathing sound
  - Heart:
    - Percussion: normal heart size
    - Ausculation: regular heart beat, normal S1, S2, no gallop, no murmur
  - Abdomen:
    - Inspection: in
      - flat contour, symetrically, no scar, no stria, no dilated veins, no rashes
    - Ausculation: normoactive bowel sound, no abdominal bruits
    - palpation: soft, no muscle guarding or rigidity, no local tenderness, no rebounding pain, no palpable mass, no palpable pulsation
    - Percussion: no shifting dulllness, no hepatomegaly, no splenomagaly
  -

Peripheral pulsation :	CA	BA	RA	FA	PA	PTA	DPA
Right side :	++	++	++	++	++	++	++/+++
Left side :	++	++	++	++	++	++	++/+++

  - Genito-anus: no tenderness or palpable mass lesion or rectal shelf, normal external genitalia, normal anal tone, brown stool

CRS CONDITION
  -
    - Lung

手術日期及方法
- 手術日期及方法20210408 1. Robotic (XI) assisted anterior resection
  2. Intraoperative colonoscopy : (-).
  3. High ligation (-) : Not reached to the IMA root. Left colic artery was preserved. IMV was ligated via robotic endoclips.
  4. Take down from the splenic flexure : (-)
  5. Robotic endoGIA (45-4.3) X2 + EEA (33mm) were applied
  6. Proximal cut end: 10 cm, distal cut end: 1 cm
  7. ADHESION BARRIER FILM was placed below the central abdominal wound
  8. No drainage tube was inserted
- 手術結果及發現 :
  - 1. A huge, 5 x 5 cm, ulcerative tumor at S-colon. 2. No obvious ascites. 3. The omentum and the small bowel over upper abdominal area was serverely adheared to each other. It was probably related to the subtotal gastrectomy procedure. 4. No obvious suspicious lesions were noted over the liver surface.

住院治療經過
- After admission, pre-op survey was done and there was no contraindication for operation. Robotic AR was performed smoothly on 04/08 and no post-op complication was noted. After OP, oral intake was smooth since 04/09 and wound was healing well. Under the stable condition, he was discharged and would be followed-up at OPD.

合併症
- nil

一般檢查
- 累積報告 :

DATE	TIME	ALB	TP	BIL.T	BIL.D	ALKP	AST	ALT	LDH	CHOL	TG	HDL-C	LDL	UA	NH3	AMY
1100409	0022	2.9		0.43	0.15		15	16								

DATE	TIME	WBC	RBC	HGB	HCT	MCV	PLT	BLST	NEUT	BAND	SEG	LYM	MON	EOS	BAS
1100409	0020	10140		11.8	37.6		297		94.4			4.0	1.3	0.1	0.2

DATE	TIME	NA	K	CL	CA	BUN	CREAT	Mg	P	CRP	ESR	PCT	LIPASE	LACTATE
1100407	1940	142	5.0											
1100409	0022	137	4.0		8.2	14	1.03							

- 放射線報告 :檢驗項目 **:CT ABDOMEN-SURVEY** 報告日期 **:1100407**
  主 訴 :
  Result:Y
  Finding:
  Imaging Report Form for Colorectal Carcinoma (AJCC 8th)

- 1. Imaging Date: 20210401
  Imaging Modality, Imaging by
  CT scan
  Imaging Protocol: CT scan of the abdomen with oral and IV contrast enhancement
  2. Tumor location / Size
  Location:
  Sigmoid
  Size:
  Measurable: at least 5.5 cm (largest diameter)
  3. Tumor invasion
  Tumor invades non-peritonealized pericolonic or perirectal tissues
  4. Regional nodal metastasis
  No
  5. Distant metastasis (In this study)
  No or Equivocal
  6. Other findings: (1). Segmental irregular wall-thickening of the sigmoid colon is noted (Ser3 Img67-76) with mild stranding of pericolic fat plane, sigmoid colon cancer may be compatible. Suggest correlation with coloscopy.
  (2). Minimal dilatation of bilateral IHDs
  (3). Multiple bilateral renal cysts
  (4). Mild enlargement of the prostate gland, suggest checking PSA level.
  (5). Focal dilatation over infra-renal abdominal aorta with mural thrombus (Ser80476 Img25), abdominal aortic aneurysm with mural thrombus may be considered.
  (6). No significant lymphadenopathy at retroperitoneum and pelvic regions
  Some small lymph nodes at subcarinal region (Ser3 Img1-5), suggest follow-up.
  (7). Patches of GGO and infiltration over bilateral visible lungs, infectious or inflammatory process may be considered.
  (8). DJD change of T- and L- spine with osteophyte formations
  Impression:
  1. R/I S-colon cancer, suggest correlation with coloscopy.
  2. For details, please see the descriptions above
- 檢驗項目 **:COLOR DOPPLER + 2D** 報告日期 **:1100407**
  檢查目的及臨床診斷：
  Result:
  TAPE NO. 7500 > BEGINS > ENDS > WT: > KG
  TAPE NO. > BEGINS > ENDS > BP: >/ > mmHG
  COMMENTS： LA (5.4 CM), RA & RV (2.9 CM) CHAMBER DILATATION
  LV (4.7, 2.6 CM) CHAMBER SIZE
  CONC. LVH (1.4, 1.5 CM), SIGMOID SEPTUM
  AORTIC ROOT DILATATION (3.9 CM)
  AORTIC VALVE THICKENED WITH MILD AR
  MODERATE MR
  MILD TO MODERATE TR WITH PEAK/MEAN SYSTOLIC PG -- 48/36 MMHG
  SUGGEST PULMONARY HYPERTENSION
  MINIMAL PR
  NORMAL LV SYSTOLIC WALL MOTION.
  THE LV EJECTION FRACTION IS 52 %
  MV E/A RATIO > 1
  ATRIAL SEPTUM ANEURYSM
  END

特殊檢查

病理報告

- Pending

其它

出院時情況

- 3.改門診治療

出院指示

藥名	劑量	劑量單位	用法	頻次	數量	數量單位	現況	天數	單位
Norvasc tab 5mg	1	TAB	PO	BID	16	TAB	TH	8	天
Dobecon tab 100mg	1	TAB	PO	TIDAC	24	TAB	TH	8	天
FinTA tab 5mg	1	TAB	PO	QD	8	TAB	TH	8	天
Hyzaar 100/12.5 mg	1	TAB	PO	QD	8	TAB	TH	8	天
(□)Takepron OD tab 30mg	1	TAB	PO	QDAC	8	TAB	TH	8	天
Mag. Oxide tab	2	TAB	PO	TID	48	TAB	TH	8	天
Ambro SR tab 75mg	1	TAB	PO	QD	8	TAB	TH	8	天
Ultracet tab	1	TAB	PO	Q6H	20	TAB	TH	5	天
Acetylcysteine tab	1	TAB	PO	BID	16	TAB	TH	8	天
Urief tab 4 mg	1	TAB	PO	BID	16	TAB	TH	8	天

出院計劃

主治醫師 2062F 蔣鋒帆 / 住院醫師 2334J 陳勇先

正式摘要(電子簽章：000000214282952)

W55 / W55

doctorRank:B
